# A Feature Fusion Based Forecasting Model for Financial Time Series

**DOI:** 10.1371/journal.pone.0101113

**Published:** 2014-06-27

**Authors:** Zhiqiang Guo, Huaiqing Wang, Quan Liu, Jie Yang

**Affiliations:** 1 Key Laboratory of Fiber Optic Sensing Technology and Information Processing, School of Information Engineering, Wuhan University of Technology, Wuhan, China; 2 Department of Financial Math and Financial Engineering, South University of Science and Technology of China, Shenzhen, China; Universidad Veracruzana, Mexico

## Abstract

Predicting the stock market has become an increasingly interesting research area for both researchers and investors, and many prediction models have been proposed. In these models, feature selection techniques are used to pre-process the raw data and remove noise. In this paper, a prediction model is constructed to forecast stock market behavior with the aid of independent component analysis, canonical correlation analysis, and a support vector machine. First, two types of features are extracted from the historical closing prices and 39 technical variables obtained by independent component analysis. Second, a canonical correlation analysis method is utilized to combine the two types of features and extract intrinsic features to improve the performance of the prediction model. Finally, a support vector machine is applied to forecast the next day's closing price. The proposed model is applied to the Shanghai stock market index and the Dow Jones index, and experimental results show that the proposed model performs better in the area of prediction than other two similar models.

## Introduction

Detecting financial time series trends is a decision support process, and stock data is typically representative of a financial time series. Two types of approaches are used to predict the stock market, namely fundamental and technical analysis. The former predicts the stock price trend by using economic factors, while the latter utilizes historical data or some technical variables to forecast the stock price. The technical analysis model can be regarded as a pattern recognition problem [1]. The model is trained using historical data or technical variables, and current data is used to predict the future stock price.

Accordingly, there are two types of stock market forecasting systems. One is to predict the stock price movement, which can be regarded as a classification problem. The other is to predict the value of the stock price, which is commonly regarded as a regression problem. For the latter, two types of forecasting frameworks exist: both auto regression and multi-variable regression models have been proposed in previous researches. The auto regression model deals with the problem using the principle of time series prediction. More specifically, the time series is divided into several segments, and then the segments are used as raw data to predict the future stock price. The basic idea of a multi-variable regression model is that related technical variables are selected as raw data to predict the future stock price.

Both auto regression and multi-variable regression models face a problem: data pre-processing. Plenty of methods are proposed to remove noise and reduce the dimensions of raw data, such as Principal Component Analysis (PCA) [2], Kernel Principal Component Analysis [3], Perpetually Important Points [4], Piecewise Aggregate Approximation[5], Singular Spectral Analysis[6], Discrete Fourier Transform, Discrete Wavelet Transform [7], the Landmarks model [8], and Random Matrix Theory[9], [65]. Zhao and Zhang [10] proposed a dimension reduction framework for time series which obtained more coefficients for recent data while fewer were kept for older data. Recently, Independent Component Analysis (ICA) has becomes a popular tool in the field of signal processing and pattern recognition, which is commonly used for feature extraction and blind signal separation.

Regarding prediction tools, some soft computing methods, such as Artificial Neural Networks (ANNs) and Support Vector Machine (SVM), have become popular methods for stock market forecasting due to their excellent nonlinear regression performance. Feed-forward Neural Networks were the first models used to detect regularities in the stock market [11]. Subsequently, Back Propagation Neural Network [12], Procedural Neural Networks [13], Probabilistic Neural Network[14], Functional Link Artificial Neural Network [15], Recurrent Neural Network [16], and Radial Basis Function Neural Network (RBFNN) [17] have been proposed for application to stock market forecasting. However, ANNs are based on the Empirical Risk Minimization principle, which may run the risk of model over-fitting and local minimums. Support Vector Regression (SVR) [18] is based on the structural risk minimization principle and has a new regression approach with good generalization ability. It has been successfully applied to problems of finance series prediction problems, which are reported in [19], [20], [21] and [22].

When modeling of financial time series using SVR, since the noise in the data could lead to over-fitting or under-fitting problems [23], data pre-processing is a key problem in this task. As a novel pre-processing tool, ICA may use higher order statistical information for separating the signals, rather than the second-order information of the sample covariance as used in PCA. ICA can therefore reveal some underlying structure in the data, giving a fresh perspective to the problem of understanding the mechanisms that influence the stock market data. Recently, a hybrid model has become popular by combining ICA and SVR in conducting time series prediction tasks. Typically, ICA and SVR are used under the auto regression framework, also known as the AICA-SVR model, such as the model in [24], [25] and [26]. ICA and SVR are used under the multivariable regression framework, also called MICA-SVR in [27], [28] and [29]. Both of them apply ICA to extract the feature from the raw data and use SVR to predict the future price. In these models, ICA and SVR are jointly employed to improve the predictive performance. However, AICA-SVR focuses on the closing price movement from the influence of the historical data, while MICA-SVR is concerned about influence from other technical variables. In fact, the stock price trend is related to both closing price history and current technical variables. In this study, a data driven model named ICA-CCA-SVR is proposed, which predicts stock closing price considering the influence of both historical closing price and current technical variables by combining ICA, Canonical Correlation Analysis (CCA), and SVR. Experimental results in the Shanghai stock market index and the Dow Jones index show that the ICA-CCA-SVR model performs better than AICA-SVR and MICA-SVR.

The article is organized as follows. In Section 2, we provide a brief explanation of the theoretical background of ICA, SVR, and the AICA-SVR and MICA-SVR models. In the subsequent section, the proposed model ICA-CCA-SVR will be explained in depth. Section 4 presents the research design and experiments, and the experimental results are presented and discussed. The final section gives the conclusion and the limitations of this study.

## Related Works

As a pre-processing tool, ICA is used in plenty of prediction models. Lu et al [20] proposed a method to predict time series using ICA as a pre-processing tool. Matteson and Tsay [30] presented an ICA for multivariable time series analysis. Ahn et al [31] used ICA as a pre-processing tool and hybrid ANNs to predict Customer Relationship Management, and the experimental result shows that the performance of ICA outperforms PCA. Mok et al [32] used ICA to extract the underlying news factors from intraday stock data to improve stock index predictions using such extracted “news factors”. Lizieri et al [33] applied an ICA procedure based on a kurtosis maximization algorithm to Real Estate Investment Trust data. The results show that ICA successfully captures kurtosis characteristics of Real Estate Investment Trust returns. Kwak et al [69] applied ICA as a dimensionality reduction tool for data mining. Lu [34] proposed an integrated independent component analysis ICA-based de-noising scheme with neural network to predict the TAIEX closing index and Nikkei 225 opening indexes. Wu and Yu [35] proposed the ICA-GARCH model which is computationally efficient in estimating the volatilities. The experimental results show that this method is more effective for modeling multivariate time series than PCA-GARCH. Cao and Chong [36] compared the performance of applications of PCA, Kernel Principal Component Analysis, and ICA to a SVM for feature extraction to predict the stock price. In these studies, a typical auto regression prediction model based on ICA and SVR is proposed by Yeh et al[26]. They regard a stock market index as a chaotic time series, and predict the index by combining ICA and SVR after phase space reconstruction. Wu and Wei [29] proposed a multivariable regression model, selecting 18 technical variables as the input of the prediction model based on ICA and SVR. In the following section, we introduce the basic idea of ICA and SVR, as well as two important prediction models, AICA-SVR and MICA-SVR.

### The principle of ICA

ICA is a tool used for the solution to the blind source separation problem. The basic idea of ICA is to extract a set of statistically independent components (ICs) from the observed signal X. Originally, ICA was used for voice signal processing and digital image processing. Later, some researchers introduced this method to finance signal analysis in order to find the independent factors hiding in the complex financial phenomenon [37].

To describe the principle of the ICA, given *m* observed signals 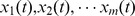, and 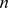 independent random signals 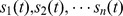, then the relationship of vector 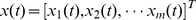 and 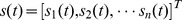 can be described as follows:
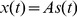
(1)or



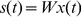
(2)Where 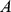 is called mixing matrix, 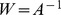 is a separation matrix, and each element 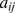 of 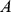 is an unknown mixture coefficient. From the formula (1), we can see each observed signal 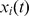 is the linear combination of the independent random signals 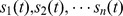. That is, 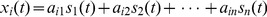
(3)the random signals 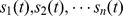 are linearly independent due to the property of statistical independence. 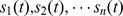 span a linear subspace, and 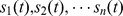 are the base of the subspace. 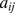 is the coefficient of a linear combination which can be regarded as the coordinates 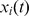 projecting the subspace 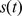. Hence, the *i*th row of matrix 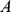 can represent the observed signal 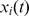 as an intrinsic feature. In general, the mixing matrix 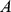 and independent components 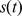 are unknown, so the basic idea of ICA is to build up an estimate model to obtain 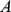, *W* and 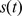 from the observed signal 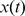, if we make an assumption that the factors are statistically independent.

Since the idea of ICA was proposed, various algorithms have been suggested to implement it, such as minimizing higher order moments [38], [39], maximization of mutual information of the outputs or maximization of the output entropy [40], minimization of the Kullback-Leibler divergence between the joint and the product of the marginal distributions of the outputs [41] and a fixed-point algorithm for ICA [42]. Among these algorithms, the fixed-point algorithm has become a very popular way to implement ICA, due to the fast convergence speed and good stability. For details of fixed-point algorithm, please refer to [43].

### The theory of SVR

SVM can be used in both classification and regression problem, the former being called a Support Vector Classifier and the latter being called a SVR. To describe the principle of SVR, given a training set 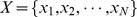, 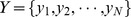, 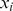 is the input of SVR, 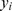 is the output of SVR. SVR approximates the function as Eq. (4)

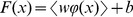
(4)where, 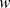 is the weight vector, 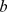 is constant, and 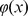 represents a kind of nonlinear function that maps *x* from the input space to the high dimensional space in order to transform the nonlinear problem into a linear one. Any function that meets Mercer's condition can be used as the kernel function such as the Gaussian kernel function, polynomial kernel function, and perception kernel function. The mathematical expressions are 
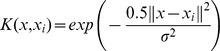
,


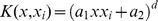
, and 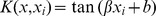, respectively. 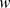 and 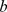 can be estimated by a minimizing function 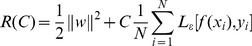
(5)where 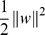 is the regularization term. Minimizing 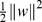 aims to control the model's capability and improve the performance of the generalization. 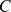 refers to a regularization constant which is used to specify the trade-off between the empirical risk and the regularization term. 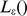 is the 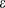 -insensitive loss function which is defined as Eq.(6)

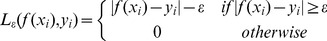
(6)where 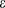 is a precision parameter which represents the tube size of the SVR. Both 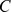 and 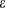 need to be per-set before the SVR is built up.

By introducing the positive slack variables 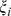 and 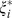, we can transform Eq. (5) into the following objective Eq. (7).
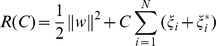



Subject to 
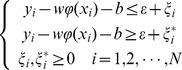
(7)


By introducing Lagrange multipliers 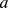, 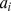 and solving the quadratic programming problem, the decision function can be expressed as Eq. (8).
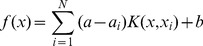
(8)where, 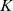 is the kernel matrix, and the element 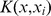 is equal to the inner product of 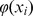 and 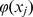 in the high dimension space, which can be computed by the kernel function.

### The auto regression model based on ICA and SVR

**Figure 1:**
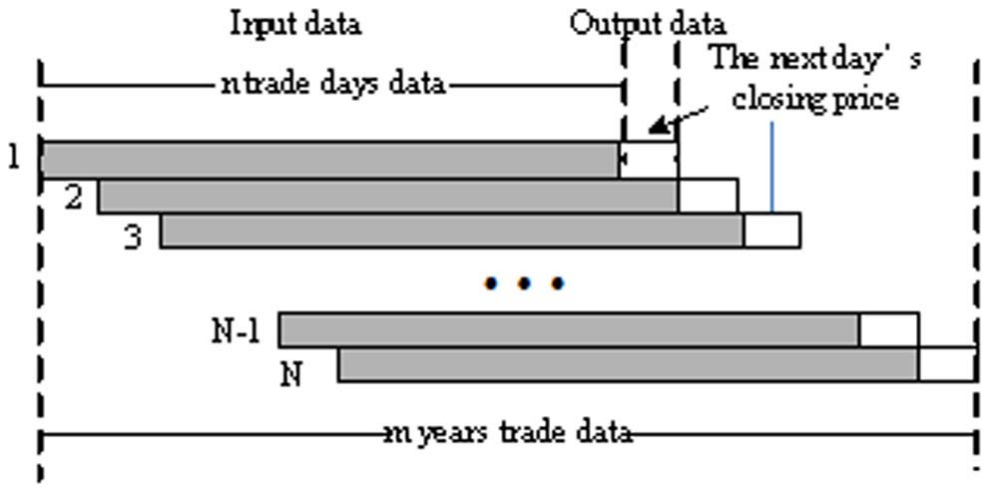
The input and output data of AICA-SVR model.

In order to obtain a return on investment, investors commonly care more about the future stock price, especially the closing price than other issues. The auto regression forecasting model is built up based on time series analysis. It aims to predict the future closing price by using the historical data. Since both the input and output are the value of the closing price, we name this type of model an auto regression model. Given a stock time series, when the slide window is moved from the beginning to the end, the training and testing samples are obtained sequentially. For example, typical input and output data of the auto regression model for stock market forecasting are shown in Fig. 1. The gray block represents the previous *n* trade days' data of stock closing price and the white block represents the *n*+1trade days' data. With the window sliding, 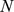 input data are obtained from the *m* year trade data set. If we want to predict the stock closing price on 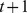 time, 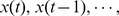 and 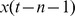 are used as the input of the model, and the output is 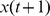. 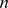 is the length of the slide window which can be selected versus empirical value.

**Figure 2:**
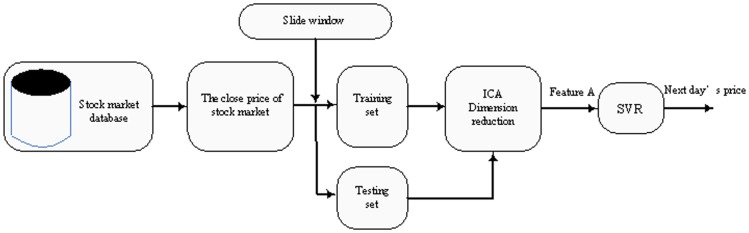
Auto ICA regression model (AICA-SVR).

Yeh et al [26] proposed the auto regression model based on ICA and SVR for time series prediction. Here, we call it the AICA-SVR model. Fig. 2 gives the stock market forecasting framework into which the model is applied. We can see that the model contains three stages: (1) the slide window is used to prepare the input data, (2) data pre-processing by ICA, and (3) forecast by SVR. The AICA-SVR model only focuses on the effect of the closing price itself, and does not pay attention to other related factors. In other words, this model behaves as if all the related factors can be reflected by the closing price of the stock, so the historical closing price decides the future trends. Actually, the movement of stock price is determined by numerous factors [44] and one single factor cannot represent all aspects needed to predict the future trends accurately.

### The multi-variable regression model based on ICA and SVR

**Figure 3:**
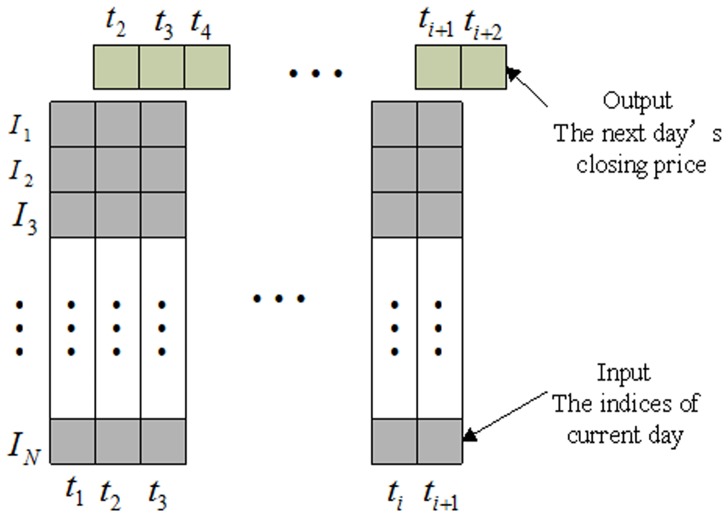
The input and output data of the MICA-SVR model.

In previous studies [45], [22], [46], researchers have believed that some technical variables could be useful for predicting price movement, such as moving averages, relative strength index, oscillator, Williams's index and so on. Based on this concept, numerous multi-variable regression models have been proposed to predict the stock price. Under this type of framework, current technical variables are selected as the input of the forecasting model and the next day's price is the output. In Fig. 3, the top row block represents the output of the model, and the low line block represents the input. For example, on the time 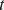, technical variables 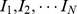 are used as the input to predict the 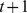 closing price 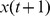. However, different models offer different variables and there is no unified measure for the selection of input technical variables [47] [48] [49]. For example, Ettes [50] selected only two input variables while Zorin and Boriso [51] used sixty-one input variables. Recently, an alternative processing method has been proposed to select sufficient variables before component analysis methods are utilized to extract the intrinsic features from them[49], [68]. After the feature is extracted, the dimension of the raw data is reduced and the noise is filtered.

**Figure 4:**
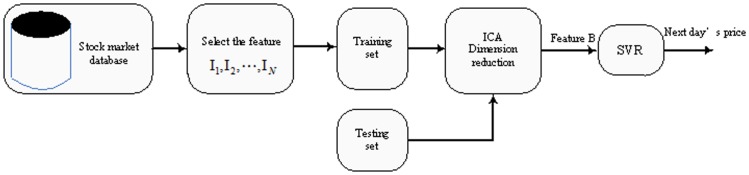
Multi-variable ICA regression model (MICA-SVR).

Lu and Wang [27], Samsudin et al [28], Wu and Wei [29] proposed the multi-variable regression model based on ICA and SVR for stock market prediction. Here, we call it MICA-SVR regression prediction model. The framework for this type of model is depicted in Fig. 4. We can see that this model contains three stages: (1) the initial exploration to prepare the technical variables, (2) dimension reduction by ICA, and (3) forecast by SVR. Compared to AICA-SVR, MICA-SVR takes some related factors into account and stresses the importance of other technical variables. However, it is not reasonable to treat the stock closing price as coequal with other technical variables. In fact, the history values of stock closing price plays the most important role in impacting the future of the closing price.

### Feature fusion

Information fusion is a new, high-level technology which collects different information from multi-sensors of the same object and removes redundant information or noise from mutual information. Commonly, there are three different fusion levels: data level fusion, feature level fusion, and decision level fusion [52]. Due to its simplicity, feature level fusion is widely used in image recognition and fault diagnosis. The basic idea of feature fusion is to extract more than one type of feature from the original data, and to combine these features by using some fusion techniques. From the point of fusion form, there are three different fusion forms: series fusion, parallel fusion, and complex vector fusion that have all been applied to various research fields [53], [54],[55],[67]. Feature fusion supplies a useful method to combine different features to a union feature for the same recognition problem. The advantage of feature fusion is that the new union feature not only keeps useful information about the original features, but also eliminates redundant information to a certain degree.

## Method

### The forecasting model based on ICA, CCA and SVR

Both the AICA-SVR and the MICA-SVR models can be regarded as pattern recognition systems. For this type of problem, the feature of the input is a key factor in impacting the prediction accuracy. AICA-SVR focuses on the closing price movement as the influence for the historical price, while MICA-SVR is more concerned about the influence of other technical variables. Both AICA-SVR and MICA-SVR have different features to deal with the same problem. It is obvious that these two types of features are both correlating and complementary.

In this paper, we propose a stock market predictive framework based on feature fusion. In this framework, an auto regression module extracts feature A from the history data of the closing price, and a multi-variable regression extracts feature B from related technical variables. The feature fusion module combines feature A and B to create a union feature by using certain fusion methods. The union feature is the input of prediction tool such as ANNs or SVR, and the output is the predicted future closing price.

**Figure 5:**
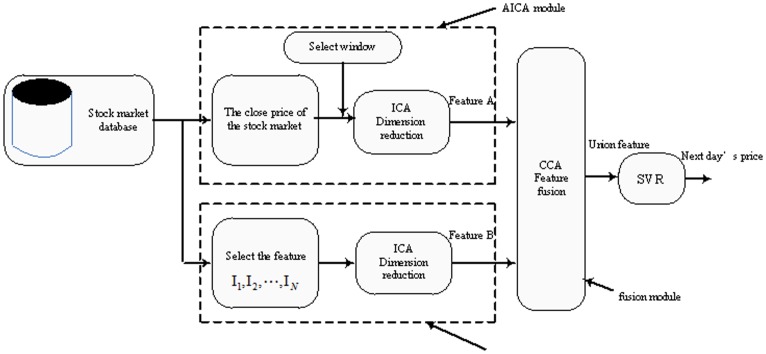
ICA-CCA regression model (ICA-CCA-SVR).

In [53], a feature fusion framework is proposed, adopting the idea of CCA for pattern recognition. Inspired by this method, we discuss a specific stock market prediction model based on a feature fusion framework. This model hybrids AICA-SVR and MICA-SVR, and utilizes CCA as the feature fusion tool to extract the union feature. SVR is used to predict the future closing price. Since this model comprehensively applies ICA, CCA and SVR approaches, we call it an ICA-CCA-SVR model. The principle of the model is displayed as Fig. 5.

The first stage of this model is that two types of features are extracted from the historical closing price and multi-variables respectively. The time series of historical closing price is divided into several segments by the slide window, then ICA is utilized to extract the first type of feature A from each segment. The length of the segment is equal to the width of the slide window. There is no definite conclusion about how to select the width of the slide window. Some studies have also indicated that recent day data have a bigger influence over the future price than old day data [56],[57]. At the same time, some researchers believe that the movement of stock price has periodicity [58], [59], so perhaps year, month and week can be used as units of length. As discussed above, we selected 30 days as the width of the slide window to produce the segments in this study.

Another type of feature B is extracted from some pre-selected technical variables by utilizing the ICA method. Thirty-nine technical variables for each day are selected as the raw data of the MICA module in this study. The name and description of the variables are displayed in Table 1. The variables are shown in publications [48],[49]. The meaning and role of the variables can be interpreted as follows. Open price, close price, high price and low price present the basic information which provides the movement of the stock market. Moving average is used to identify the direction of the price trend. BIAS serves as an indicator of overbought or oversold conditions and an indicator of price breakouts. Exponential moving average returns the exponential moving average of the specified period. Moving average convergence divergence displays trend following characteristics and momentum characteristics. The stochastic oscillator measures how much a price tends to close in the upper or lower areas of its trading range. Price rate of change shows the speed at which a stock's price is changing. True range returns a numeric value containing the difference between the true high and true low of the price. Momentum can help pinpoint the end of a decline or advance. Williams index Uses Stochastics to determine overbought and advance. Oscillator shows how a stock's price is doing relative to past movements. Relative Strength Index shows how strongly a stock is moving in its current direction. Phychological line reflects the buying power in relation to the selling power. On balance volume combines price and volume to show how money may be flowing into or out of a stock. Bollinger band shows the upper and lower limits of normal price movements based on the standard deviation of prices. Emotional index is the most important index to measure the power changes of the straddle both sides. AR index called popular indicators, BR index called the sale will target, they are both long and short measure of market forces the most important indicators of change. Other variables (e.g. I_5_, I_12_, I_33_ to I_39_) reflect the change of close price, exponential moving average, stochastic %K %D and moving average. More detail about the technical indicators please refer to [64].

**Table 1:** Variables used as inputs.

name	Description or Formula
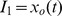	Open price
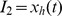	High price
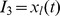	Low price
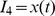	Close price
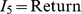	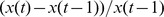
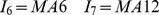	Moving average
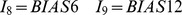	BIAS
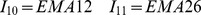	Exponential Moving Average
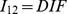	EMA12-EMA26
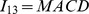	Moving Average Convergence Divergence
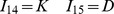	Stochastic %K %D
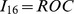	Price rate of change
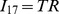	True range of price Movements
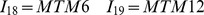	Momentum
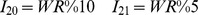	Williams index
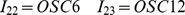	Oscillator
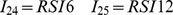	Relative strength index
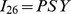	Phycholoigical Line
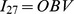	On Balance Volume
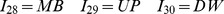	Boll line
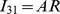	A ratio
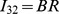	B ratio
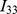	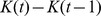
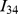	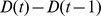
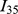	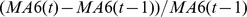
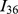	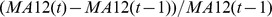
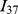	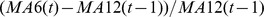
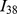	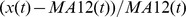
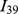	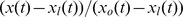

In order to extract features A and B, the historical data and technique variables data are organized to reshape the observed data 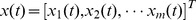 according to Figs 1 and 2, respectively. Then, a fixed-point algorithm is used to generate mixing matrix 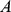 and independent components S(*t*) in formula (1). The *i*th row 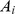 of matrix 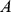 is regarded as the ICA feature of the observed data *x_i_*(*t*). If is generated from historical data of the stock price, then 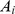 is feature **A**; if *x*(*t*) is generated from technique variables data, then 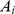 is feature **B**.

In the ICA algorithm, the selection of ICs subspace is a key issue. Bartlett et al [43] have proposed three methods to tackle this problem: (1) Method based on an amplitude of weight vector; (2) PCA-ICA method; and (3) Scaling factor method based on cluster analysis. Method (2) depends heavily on the PCA method and the selected subspace is kept within the PCA subspace. Method (3) is suitable to the classification problems but not regression problems, so Method (1) based on amplitude of weight vector is selected to reduce the dimension of raw data in this study.

The second stage of the model is the fusion module. CCA is used in this model to be the fusion tool. Hotelling [60] developed CCA, which is used to analyze the correlation problem of two random vectors. Suppose that 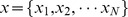 and 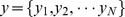 represent the feature **A** and **B** extracted from the AICA module and the MICA module, respectively. 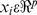 and 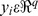 are the features of the ith sample. The basic idea of CCA is to find two project directions 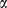 and 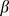 to maximise the correlation of 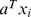 and 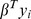 while minimizing the correlation between the elements of 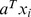 and 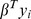. Pearson Correlation coefficient can be used to measure the relationship between 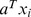 and 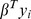,we expect to search for the optimal values 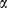 and 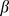 and maximize correlation 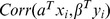,so the following objective function is given to solve the problem,
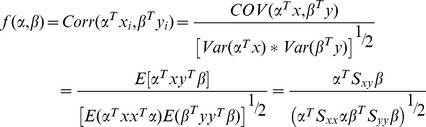
(9)



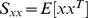
 and 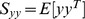 are the covariance matrices of *x* and *y* respectively, while 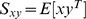 denotes the between covariance matrix of *x* and *y*. Given the constrain
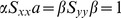
(10)by introducing Lagrange multipliers 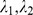, the objective function (9) can be transformed to maximize the following equation



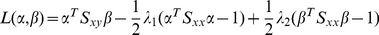
(11)The partial derivatives of 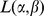 with respect of 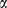 and 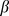 are then equalled to zero respectively.



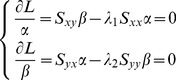
(12)multiplying both side of each Eq. (12) by 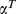 and 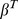, respectively. 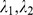 can be obtained by 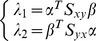. Consider 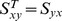, then 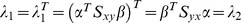, and let 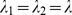, we can obtain the relationship of 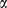 and 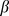.
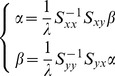
(13)


Substituting Eq. (13) into Eq. (12), obtain the following eigenfunction
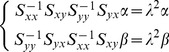
(14)where 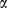 and 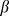 are the eigenvector of eigenfunction, respectively. The projection matrix 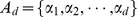 and 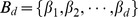 is composed of the eigenvectors corresponding to the first d largest eigenvalues of function (15). After 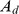 and 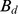 are calculated, the fusion feature of 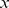 and 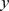 can be obtained by the following equation.



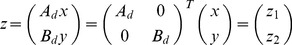
(15)In order to solve Eq. (14), 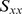 and 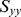 must be nonsingular. If 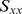 and 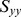 are singular, we can use the perturbation method (Hong 1999) to modify 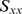 and 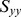. The main idea is that a small perturbation is added to the singular 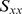 and 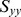 such that 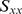 and 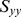 becomes nonsingular, i.e. full rank matrix. For more details, please refer to [66].

The third stage of the model is the SVR used to predict the future value of the closing price. After the fusion feature is obtained, it can be used to train the stock market prediction model based on SVR. Its first step is to choose the kernel function. Different kernel function may yield different performances. Research indicates that the Gaussian kernel function shows good performance for forecasting problems [20]. Therefore, as it is suitable to cope with the finance series prediction problem, we choose the Gaussian kernel function for SVR. Another key problem of the SVR is to decide the parameters 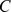, 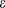 and 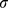 which will deeply affect the predictive performance. The selection of 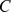, 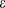 and kernel parameter 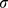 is an open problem, and a cross-validation method is commonly used in some research fields.

### Model analysis

In this section, we give an intuitive analysis for the ICA-CCA-SVR model. The advantage of the proposed model may lie on the following reasons.

First, as a component analysis tool, ICA is often compared to another popular component analysis tool, PCA. The first difference between ICA and PCA is that the components of PCA are orthogonal, while those of ICA are independent. Secondly, PCA can only extract second order statistic characteristics of the observed signal. However, ICA can obtain the high order statistic characteristic hiding in the signal. Moreover, PCA application to the signal analysis is that the original data should be satisfied with a normal distribution. Unfortunately, this condition cannot be satisfied in a practical application such as stock market analysis. ICA does not demand that the original data follow a normal distribution.

Second, the main characteristic of ICA-CCA-SVR is that it combines two types of features that are used in the AICA-SVR and MAICA-SVR models. The advantage of this structure is the following: (1) From the feature extraction principle of ICA, the feature in the AICA-SVR and MAICA-SVR models defines the coordinates that the raw data projects into the ICA subspace; that is, the row vectors of mixing matrix *A*. In ICA subspace, the base vectors are independent because they represent the independent component of the raw data. However, from Eq. (3), we can see that the coefficients of linear combination are not independent, indicating that the features in the AICA-SVR and MAICA-SVR models are not independent. In this case, the features in both models must have redundant information. (2) Features in AICA-SVR and MAICA-SVR are the description for the same predictive object. It is obvious that common information between the two features is the most important factor for predictive performance. That is, two types of features have a certain correlation. (3) Features in AICA-SVR and MAICA-SVR are extracted from different observed points for the same predictive object. The former pays attention to the influence of the history data of the predictive object, and the latter focuses on related factors outside the predictive object. Thus the two types of feature have mutual complementarity. Based on the discussion above, it is quite useful for the improvement of the predictive performance to wipe off redundant information and combine the two types of features. From section 3.1, the union feature of the ICA-CCA-SVR model is made up of two parts 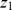 and 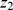. Based on the principle of CCA, the elements of 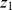 and 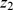 have minimum correlation; compared to the original 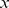 and 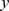, 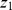 and 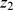 have lower information redundancy. At the same time, there is maximum correlation between 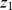 and 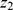, as serial features fusion strengthens the predictive information.

Finally, compared to ANNs and Basis Function Neural Network, SVR based on structural risk minimization has a stronger generalization capability to tackle the stock market forecasting problem. A cross-validation method is used to select the SVM parameters, which makes the model have better adaptability.

We give a computational complexity analysis of the three models. If the dimension of original features of AICA-SVR and MAICA-SVR is 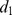 and 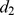, respectively, the training sample is 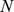, the dimension of feature **A** and feature **B** is 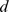, and 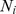 is the maximum iteration of the ICA algorithm, then the computational complexity of AICA, MICA, SVR and CCA are 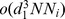, 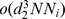, 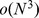, and 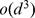, respectively. So the computational complexity of AICA-SVR is 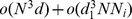, the computational complexity of MICA-SVR is 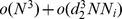, and the computational complexity of ICA-CCA-SVR is 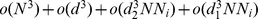. In the proposed model, 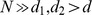, (e.g. 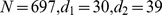 and 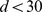 the case study on the Shanghai stock marke) so the computational complexity of the three models depends on 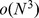. That is, the computational complexities of the three models have the same order of magnitude.

## Experiments

To evaluate the performance of the ICA-CCA-SVR model, we performed experiments on two real-world datasets: the Shanghai stock market index and the Dow Jones index. Comparison was made with the AICA-SVR and the MICA-SVR models. We performed experiments on a PC with Intel (R) Core (TM) i3 CPU, 2G RAM memory, on a MATLAB 7.0 platform.

### Data set description

The Shanghai stock market index data collected from January 4, 2003 to December 31, 2005 are used in this experiment. The overall data includes 1180 trading days' data, which are split into two parts: January 4, 2003 to December 31, 2004 and January 1, 2005 to December 31, 2005. The former, which includes 726 trading days' data, is used as the training set, and the latter, which includes 242 trading days' data, is used as the testing set.

To test the robustness of the model, we selected three years' worth of data from the Dow Jones index, which includes two years of data for the training set and one year of data for the testing set. The Dow Jones index data were collected from January 2, 2003 to December 31, 2005 for use in this experiment. The overall data includes 1260 trading days of data, which are split into two parts: January 2, 2003 to December 31, 2004 and January 1, 2005 to December 31, 2005. The former, which includes 507 trading days' data, is used as the training set, and the latter, which includes 252 trading days' data, is used as the testing set.

### Experimental settings measure index selection

As discussed in Section 3, the length of the slide window is 30 days so as to build up the raw data of the AICA module, and the 39 technical variables in Table 1 are used for the raw data of the MICA module. The forecasting performance of the proposed model ICA-CCA-SVR is compared to those of the AICA-SVR and MICA-SVR models. AICA-SVR uses previous price to predict the next days' price, while MICA-SVR uses current technical variables, and ICA-CCA-SVR uses both previous price and current technical variables for its prediction. To build the three models discussed above, we use the libsvm toolbox to compute the SVM algorithm, which is compiled by Chih-Jen Lin, a professor at Taiwan University (http://www.csie.ntu.edu.tw/~cjlin/). Cao and Tay [56] showed that SVR are insensitive to ε, as long as it is a reasonable value. Therefore, we choose 0.01 for 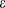 in all the experiments in this study. In determining the kernel bandwidth 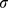 and the margin 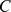, a three-fold cross validation technique is used to choose parameters that yield the best results, where 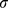 and 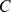 range from 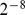 to 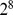, the varying exponent step is selected as 1. ICs are ordered by the method based on amplitude of weight vector.

The predictive performance is evaluated by using the following performance measures, namely, Correlation Coefficient (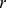), Non-linear Regression Multiple Correlation Coefficient (R^2^), Mean Absolute Error (MAE), Mean Absolute Percentage Error (MAPE), Mean Squared Error (MSE),and Root Mean Square Error (RMSE) [61],[62],[63]. The description and formulae of these indicators are given in columns 2 and 3 in Table 2. These indicators are used to measure whether the predicted value is similar to the actual value. If 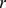 and R^2^ are bigger, it means that the predicting value is similar to the actual value. If MAE, MAPE, MSE, and RMSE are smaller, this also indicates that the predicted value is close to the actual value. In the table, 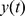 and 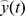 represents the actual value and predicted value respectively.

**Table 2:** Measure indicators.

name	Description	Formula
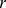	correlation coefficient	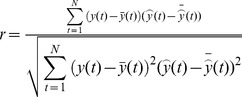
**R^2^**	Non-linear regression multiple correlation coefficient	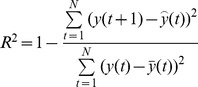
**MAE**	Mean absolute error	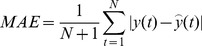
**MAPE**	Mean Absolute Percentage Error	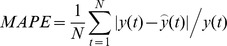
**MSE**	Mean squared error	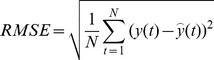
**RMSE**	Root mean square error	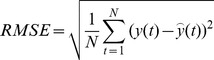

### Experimental result

As we have discussed in Section 2, the Selection of ICs is the key issue in the data pre-processing. Different numbers of ICs correspond to the different dimensionality of features A and B, which have a strong influence on the predictive performance of the models. To determine the dimensionality of features A and B in our framework, we compare the correlation coefficient 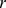 as we change the dimensionality 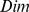 in isolation. For the AIC-SVR model, since the dimensionality of raw data is 30, the dimensionality of feature A is ranged from 1 to 29. In order to compare the AICA-SVR model, the dimensionality of feature B is also selected from 1 to 29.

**Figure 6:**
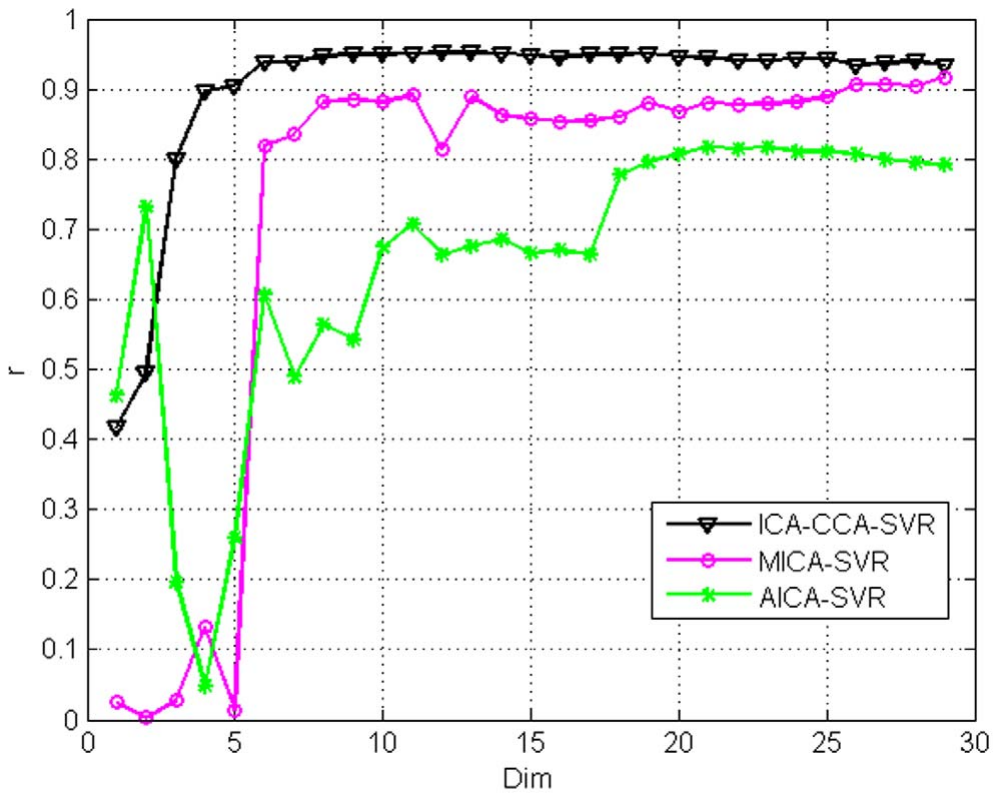
Shows the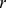 curve versus the variation of dimensions and the proposed method consistently outperforms the other methods in the Shanghai stock market index.

**Figure 7:**
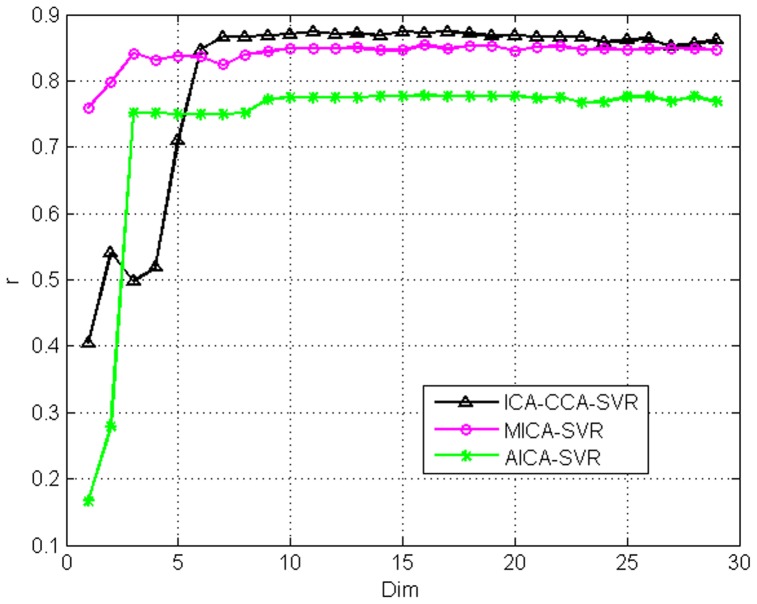
Shows the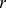 curve versus the variation of dimensions and the proposed method consistently outperforms the other methods in the Dow Jones index.

The curves between 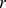 and 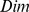 on the Shanghai stock index and Dow Jones index are displayed in the Figs. 6 and 7, respectively. From these figures, we can not only find the optimal dimensionality, but also illustrate the validity of the selected features of the three models. Figs. 6 and 7 give a comparison in terms of 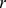 using AICA-SVR and MICA-SVR, and ICA-CCA-SVR, respectively. The number 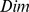 in the X-axis of figures refers to the dimensionality of features using different feature selection models. Note that the actual dimensionality of the ICA-CCA-SVR model is 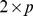 if the dimensionality of the MICA-SVR and AICA-SVR models are p based Eq.(15). For convenience, we draw the curves between *r* and 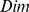 for the three models in a single figure. From the curves in Figs. 6 and 7, we can see that the AICA-SVR model's amplitude of fluctuation is the largest among all models, indicating that the AICA-SVR model is not as stable as the other two models. One possible underlying reason is that the single variable does not contain sufficient information whereas the multi-variable does. On the Shanghai stock market index, when dimensionality is smaller than 5, the 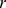 of the AICA-SVR model is higher than that of the MICA-SVR model. However, when the dimensionality is more than 8, the 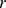 of the MICA-SVR model is much higher than that of AICA-SVR. The highest 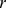 of the MICA-SVR model is 0.91685, which is also much bigger than that of AICA-SVR's 0.8174. On the Dow Jones index, the 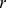 of the MICA-SVR model is higher than that of AICA-SVR in the whole range of the dimensionality.

The performance of the ICA-CCA-SVR model is superior to both MICA-SVR and AICA-SVR with the increase of 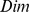. On the Shanghai stock market index, all plots of the ICA-CCA-SVR model are higher than MICA-SVR and AICA-SVR, although the first two plots have no distinct advantages and are even lower than AICA-SVR, due to the prediction information not being sufficient at lower dimensionality. The highest *r* of the ICA-CCA-SVR model is 0.95174, which is also much bigger than that of AICA-SVR and MICA-SVR. At the same time, as the 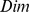 increases, the ICA-CCA-SVR model shows stable predictive performance compared to the other models. On the Dow Jones index, when dimensionality is smaller than 5, the 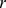 of the ICA-CCA-SVR model is lower than that of the MICA-SVR model, and even is lower than AICA-SVR when 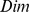 is equal to 3, 4 and 5. However, when the dimensionality is increased to 6, the *r* of the ICA-CCA-SVR model is much higher than that of AICA-SVR and MICA-SVR. The ICA-CCA-SVR model obtains the highest *r* of 0.87446 among the three models.

**Figure 8:**
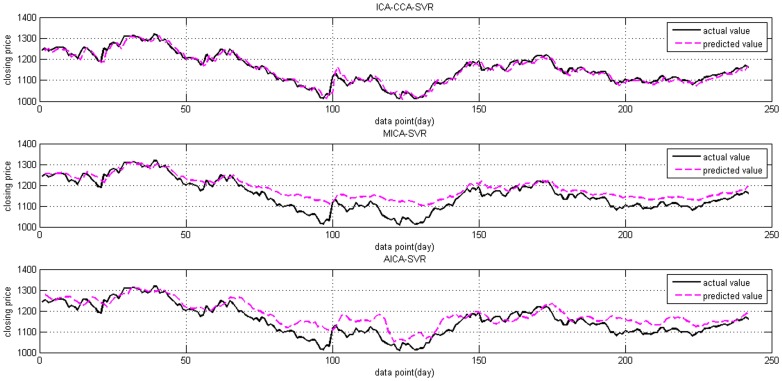
The actual Shanghai stock market index and its predicted values from ICA-CCA-SVR, MICA-SVR, and A ICA-SVR.

**Figure 9:**
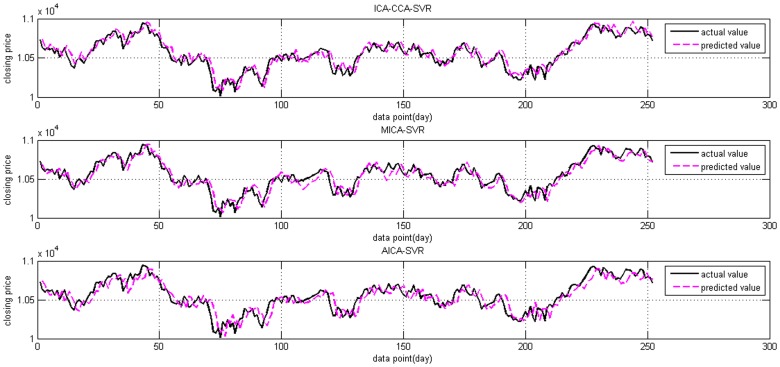
The actual Dow Jones index and its predicted values from ICA-CCA-SVR, MICA-SVR, and AICA-SVR.

The actual Shanghai stock market index and predicted values from all three models are illustrated in Fig. 8 and Fig. 9 is the same curve for the Dow Jones index. It can be observed from Fig. 8 that the predicted values obtained from the proposed ICA-CCA-SVR model are closer to the actual values than those of the MICA-SVR and AICA-SVR models. From Fig. 9, we can see that the predicted values on the Dow Jones index of all three models are not fitted as well as they are on the Shanghai stock market index. Even so, the ICA-CCA-SVR model remains superior to the other two models.

For comparison, AICA-RVR and MICA-SVR model were applied to evaluate the prediction accuracy of the proposed ICA-CCA-SVR model. Table 3 and Table 4 show the prediction results for AICA-SVR, MICA-SVR and the proposed ICA-CCA-SVR models. In the Tables, Dim, BestC, and Bestg represent the optimal dimensionality, parameter C, and parameter σ for each model respectively. The comparison results show that the proposed ICA-CCA model has the smallest RMSE, MAPE, MSE and MAE values, and the highest R^2^, and 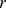 values in comparison with MICA-SVR and AICA-SVR. Table 3 demonstrates the comparisons of the forecasting results of three models for Shanghai stock market index. It can be seen from the table that the ICA-CCA-SVR model shows much better performance than the other two models. All the measure indicators of the ICA-CCA-SVR model are significantly improved after feature fusion. For example, the indicators MAPE, RMSE, MAE and MSE values of the ICA-CCA-SVR model reach 0.011, 16.54, 12.638 and 273.56 respectively, which is much less than those of the AICA-SVR and MICA-SVR models. The indicators R^2^ and 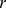 values of ICA-CCA-SVR model reach 0.9486 and 0.95174, which is much bigger than those of the AICA-RVR and MICA-SVR models. Comparing the MICA-SVR model with the AICA-SVR model, the MICA-SVR model shows better performance than the AICA-SVR model. Table 4 compares the forecasting results derived from the three models for Dow Jones index, we can see that the three models do not work as well as they do in the Shanghai stock market. The result shows that the proposed model also has the lowest MAPE, RMSE, MAE and MSE and the highest R^2^ and 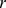 values and outperforms the AICA-SVR and MICA-SVR models. It concludes that the proposed ICA-CCA-SVR model can produce lower prediction errors and higher prediction accuracy in the direction of change in price and outperforms MICA-SVR and AICA-SVR methods in forecasting the Shanghai stock market index and Dow Jones index closing prices.

**Table 3:** Measure index on the set of Shanghai stock market index.

Method	Dim	Bestc	Bestg	MAPE	RMSE	MAE	R^2^	MSE	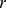
ICA-CCA-SVR	12	8	0.03125	**0.011**	**16.54**	**12.638**	**0.9486**	**273.56**	**0.95174**
MICA-SVR	29	16	0.0625	0.0308	42.274	33.896	0.33861	1787.1	0.91685
AICA-SVR	23	32	0.0625	0.0316	43.667	35.133	0.43969	1906.8	0.8174

**Table 4:** Measure index on the set of Dow Jones index.

Method	Dim	Bestc	Bestg	MAPE	RMSE	MAE	R^2^	MSE	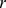
ICA-CCA-SVR	15	4	0.03125	**0.0056**	**72.549**	**58.249**	**0.85951**	**5263.4**	**0.87446**
MICA-SVR	16	16	0.03125	0.0058	76.58	60.61	0.8391	5864	0.85338
AICA-SVR	16	32	0.125	0.0071	93.559	73.896	0.7276	8753.3	0.77746

## Discussion

From the above results, we can draw the conclusion that the ICA-CCA-SVR model performs well and surpasses the AICA-SVR and MICA-SVR models. The predicted stock price is influenced both by its the historical price and by related technical variables. However, both the AICA-SVR and MICA-SVR models only extract the features from one side. The ICA-CCA-SVR model further removes redundant information from AICA and MICA features, and combines the retained useful information to improve predictive performance. We also notice that the performance of ICA-CCA-SVR is no better than that of AICA-SVR and MICA-SVR models, when the projecting dimensionality is low, that is, less than 3 for the Shanghai stock market index and 6 for the Dow Jones index. We believe that the possible reasons lie in the two following explanations. (1) The lower the dimensionality, the greater the ratio of noise to useful information is contained in the features. In this case, the fusion feature will strengthen the noise to impact the predictive performance. (2) For the CCA algorithm, the component of extracted features is uncorrelated but not independent, which means that the components have no influence over each other in the sense of statistical average. However, it cannot be visibly displayed when the components are insufficient.

## Conclusion

This paper builds a forecasting model to predict the closing price of the stock market. It utilizes ICA and CCA as tools to extract predictive features before constructing an SVR stock market forecasting model. Experimental results on the Shanghai stock market index and on the Dow Jones index show that the ICA-CCA-SVR model proposed in this paper obtains better performance than both the AICA-SVR and MICA-SVR models.

Noise and redundant information exist in original stock market data, so feature extraction is a vital step in a forecasting model. Various types of existing forecasting models only emphasize the classifier of the model and pay little attention to the pre-processing of the data. In this study, we introduce ICA as the pre-processing tool to reduce the dimensions and to extract features from two different points. CCA is used as feature fusion tool to extract the intrinsic features of the input raw data. Due to the fusion feature extraction characteristic, ICA-CCA shows better performance in evaluating stock market data.

Although the proposed model provides many insights, it also has minor weaknesses. The forecasting accuracy of the model is not particularly high; for example, the highest correlation coefficient is 0.95174 for the Shanghai stock market index and 0.87446 for the Dow Jones index. We believe the main reason is that the proposed model has a certain sensibility to the data. Another weakness is that the optimal feature dimensionality of the ICA-CCA-SVR model may sometimes be higher than that of the other models, due to the serial features fusion method. To solve this problem, utilization of a more effective method as the parallel fusion method will be investigated through further study.

## References

[1] Felsen J (1975) Learning pattern recognition techniques applied to stock market forecasting. IEEE T SYS MAN CY 5(6): 583–594.

[2] Verleysen M, François D (2005) The curse of dimensionality in data mining and time series prediction.In Proceeding of the 18^th^ International Conference on Artificial Neural Networks:computational Intelligence and Bioinspired Systems, Springer-Verlag Berlin, Heidelberg, pp 758–770.

[3] Ince H, Trafalis T (2007) Kernel principal component analysis and support vector machines for stock price prediction. IIE Transactions 39(6): 629–637.

[4] Fu T, Chung F, Ng V, Luk R (2001) Pattern discovery from stock time series using self-organizing maps. In Workshop Notes of KDD2001 Workshop on Temporal Data Mining, pp 27–37.

[5] Keogh E, Chakrabati K, Pazzani M, Mehrota S (2000) Dimensionality reduction for fast similarity search in large time series databases. Knowledge and Information Systems 3(3): 263–286.

[6] Bunau PV, Meinecke FC, Kiraly FC, Muller KR (2009) Finding stationary subspaces in multivariate time series Physical review letters. 103(21): 214101. 10.1103/PhysRevLett.103.214101 20366040

[7] Batal I,Hauskrecht M (2009) A supervised time series feature extraction technique using DCT and DWT. In International Conference on Machine Learning and Applications, Miami Beach, Florida, pp 735–739.

[8] Perng CS, Wang H, Zhang SR, Parker DS (2000) Landmarks: A new model for similarity-based pattern querying in time series databases. In 16 International Conference on Data Engineering, IEEE ICDE, pp 33–42.

[9] Laloux L, Cizeau P, Bouchaud JP, Potters M (1999) Noise dressing of financial correlation matrices. Phys. Rev. Lett 83(7): 1467–1470.

[10] Zhao Y, Zhang S (2006) Generalized dimension-reduction framework for recent-biased time series analysis. IEEE Trans Knowl Data Eng 18(2): 231–244.

[11] White H (1988) Economic prediction using neural networks: A case of IBM daily stock returns. In IEEE International Conference on Neural Networks, pp 451–458.

[12] Sutheebanjard P, Premchaiswadi W (2010). Stock exchange of Thailand index prediction using back propagation neural networks. In 2nd International Conference on Computer and Network Technology. IEEE Computer Society Press, Washington, pp 377–380.

[13] Liang J, Song W, Wang M (2011) Stock market prediction based on procedural neural networks. Advances in Artificial Neural Systems doi:10.1155/2011/814769

[14] Chen AS, Leung MT, Daouk H (2003) Application of neural networks to an emerging financial market: Forecasting and trading the Taiwan Stock Index. Comput Oper Res 30(6): 901–923.

[15] Padhiary PK, Mishra AP (2011) Development of improved artificial neural network model for stock market prediction. Int J Eng Sci 3(2): 1576–1581.

[16] Hsieh TJ, Hsiao HF, Yeh WC (2011) Forecasting stock markets using wavelet transforms and recurrent neural networks: An integrated system based on artificial bee colony algorithm. Appl Soft Comput 11(2): 2510–2525.

[17] Parras-Gutierrez E, Garcia-Arenas M, Rivas VM, Jesus MJ (2012) Coevolution of lags and RBFNs for time series forecasting: L-Co-R algorithm. Soft Comput 16(6): 919–942.

[18] Vapnik VN (1995) The Nature of Statistical L earning Theory, Springer-Verlag, New York.

[19] Das SP, Padhy S (2012) Support vector machines for prediction of futures prices in Indian stock market. Int J Comput Appl 41(3): 22–26.

[20] Lu CJ, Lee TS, Chiu CC (2009) Financial time series forecasting using independent component analysis and support vector regression. Decision Support Systems 47 (2): 115–125.

[21] He W, Wang Z, Jiang H (2008) Model optimizing and feature selecting for support vector regression in time series forecasting. Neurocomputing 72 (1–3): 600–611.

[22] Huang W, Nakamori Y, Wang SY (2005) Forecasting stock market movement direction with support vector machine. Comput Oper Res 32(10): 2513–2522.

[23] Cao LJ (2003) Support vector machines experts for time series forecasting, Neurocomputing. (51): 321–339.

[24] Huang SC, Li CC, Lee CW, Chang MJ (2010) Combining ICA with kernel based regressions for trading support systems on financial options. In The 3rd International Symposium on Intelligent Decision Technologies and Intelligent Interactive Multimedia Systems and Services. Baltimore, pp 163–169.

[25] Guo C, Su M (2001) Spectral clustering method based on independent component analysis for time series. Systems Engineering Theory & Practice 31(10): 1921–1931.

[26] Yeh CC, Chang B, Lin HC (2009).Integrating phase space reconstruction, independent component analysis and random forest for financial time series forecasting. In The 29th Annual International Symposium on Forecasting, Hong Kong, pp 21–24.

[27] Lu CJ, Wang YW (2010) Combining independent component analysis and growing hierarchical self-organizing maps with support vector regression in product demand forecasting. Int.J. Production Economics 128(2): 603–613.

[28] Samsudin R, Shabri A, Saad P (2010) A comparison of time series forecasting using support vector machine and artificial neural network model. Journal of Applied Sciences 10(11): 950–958.

[29] Wu JX, Wei JL (2007) Combining ICA with SVR for prediction of finance time Series. In IEEE International Conference on Automation and Logistics. Jinnan, pp 18–21.

[30] Matteson DS, Tsay RS (2011) Dynamic orthogonal components for multivariate time series. J AM Stat Assoc 160 (496): 1450–1463.

[31] Ahn H, Choi E, Han I (2007) Extracting underlying meaningful features and canceling noise using independent component analysis for direct marketing. Expert Syst Appl 33(1): 181–191.

[32] Mok PY, Lam KP, NG HS (2004) An ICA design of intraday stock prediction models with automatic variable selection, IEEE International Joint Conference on Neural Networks, Vol 3,Budapest, pp 2135–2140.

[33] Lizieri C, Satchell S, Zhang Q (2007) The underlying return-generating Factors for REIT Returns: an application of independent component analysis. Real Estate Economics 35(4): 569–598.

[34] Lu CJ (2010) Integrating independent component analysis-based denoising scheme with neural network for stock price prediction. Expert Syst Appl.37 (10): 7056–7064.

[35] Wu E, Yu P (2005) Volatility modelling of multivariate financial time series by using ICA-GARCH models. Intelligent Data Engineering and Automated Learning - IDEAL 2005 Lecture Notes in Computer Science (3578/2005): 179–187.

[36] Cao LJ, Chong WK (2003) A comparison of PCA, KPCA and ICA for dimensionality reduction in support vector machine. Neurocomputing 55(1–2): 321–336.

[37] Hyvarinen A, Oja E (2000) Independent component analysis: algorithms and applications. Neural Networks 13(4–5): 411–430. 10946390 10.1016/s0893-6080(00)00026-5

[38] Cardoso J (1989) Source separation using higher order moments, International Conference on Acoustics, Speech and Signal Processing, Vol 4, Glasgow, pp 2109–2112.

[39] Cardoso J, Souloumiac A (1993) Blind beamforming for non-Gaussian signals. Radar and Signal Processing, IEE Proceedings F 140(6): 771–774.

[40] Bel A, Sejnowski T (1995) An information maximization approach to blind separation and blind deconvolution. Neural Computation 7(6): 1129–1159. 7584893 10.1162/neco.1995.7.6.1129

[41] Amari S, Cichocki A, Yang H (1996) A new learning algorithm for blind signal separation, in Advances in Neural Information Processing Systems 8, Editors D Tesauro, M Mozer and M Hasselmo, MIT Press, Cambridge, pp 757–763.

[42] Hyvarinen A (1996) A Simple one-unit algorithms for blind source separation and blind deconvolution,In Proceeding of International Conference on Neural Information Processing, Vol 2, Hong Kong, pp 1201–1206.

[43] Bartlett MS, Movellan JR, Sejnowski TJ (2002) Face Recognition by Independent Component Analysis, IEEE Trans. on Neural Networks 13(6): 1450–1464. 18244540 10.1109/TNN.2002.804287 PMC2898524

[44] Yu L, Chen HH, Wang SY, Lai KK (2009) Evolving Least Squares Support Vector Machines for Stock Market Trend Mining. IEEE Transactions on evolutionary computation. 13(1): 87–102.

[45] Kim KJ (2003) Financial time series forecasting using support vector machines. Neurocomputing 55 (1–2): 307–319.

[46] Sui XS, Qi ZY, Yu DR, Hu QH, Zhao H, et al. (2007) A Novel Feature Selection Approach Using Classification Complexity for SVM of Stock Market Trend Prediction. International Conference on Management Science & Engineering (14th). Harbin, pp1654–1659.

[47] Atsalakis GS, Valavanis KP (2009) Surveying stock market forecasting techniques – Part II: Soft computing methods. Expert Systems with Applications 36(3): 5932–5941.

[48] Teixeira LA, Oliveira ALI (2010) A method for automatic stock trading combining technical analysis and nearest neighbor classification. ?Expert Syst Appl 37(10): 6885–6890.

[49] Huang W, Nakamori Y, Wang SY (2005) Forecasting stock market movement direction with support vector machine. Comput. Operat. Res 32(10): 2513–2522.

[50] Ettes D (2000) Trading the stock markets using genetic fuzzy modelling. In Proceedings of Conference on Computational Intelligence for Financial Engineering, pp 22–25.

[51] Zorin A, Boriso A (2002) Modelling riga stock exchange index using neural networks. http://www.knolsearch.com/ebook/-F0eT3uE5qwfgttFnFZIS8doW2xnVMqs6sJfXBQ_UAU00XDpkOMQPg70XrJTtTAx9dvs3garSmzN_OvNYyTY0napVPSp6RTXA6Z3aq0Ntwrk_zl526VtGmqHZwFDRRh0ClOin1_mfNy92a3Gk9plNbtHbFnr10m-9-kvSUa50MB4/modelling-riga-stock-exchange-index-using-neural-networks-…-LASG.html.

[52] Hall DL, Linas J (1997) An introduction to multisensory data fusion. Proceedings of the IEEE 85(1): 6–23.

[53] Sun QS, Zeng SG, Liu Y, Heng PA, Xia DS, et al (2005) A new method of feature fusion and its application in image Recognition. Pattern Recognition 38(12): 2437–2448.

[54] Cui B, Anthony KH, Zhang TC, Zhao Z (2010). Multiple feature fusion for social media applications. Proceedings of the 2010 ACM SIGMOD. Indianapolis, pp 435–446.

[55] Su SZ, Chen SY (2013) Analysis of feature fusion based on HIK SVM and its application for pedestrian detection. Abstract and Applied Analysis. Vol.2013, pp.1–11, Online: http://dx.doi.org/ doi:10.1155/2013/436062

[56] Cao LJ, Tay FEH (2003) Support vector machine with adaptive parameters in financial time series forecasting. IEEE Trans Neural Netw 14(6): 1506–1518. 18244595 10.1109/TNN.2003.820556

[57] Tay FEH, Cao LJ (2002) Modified support vector machines in nancial time series forecasting. Neurocomputing 48(1–4): 847–861.

[58] Cowles A, Jones HE (1937) Some a posteriori probabilities in stock market action. The econometric society 5(3): 280–294.

[59] Dong Y, Yang BC (2006) The periodic analysis of realized volatility in china stock market. Journal of Tianjin University of Technology 22(6): 24–26.

[60] Hotelling H (1936) Relations between two sets of variates. Biometrika.28 (3–4): 321–377.

[61] Kai KA, Chai Q (2006) Stock Trading Using RSPOP: A Novel Rough Set-Based Neuro-Fuzzy Approach. IEEE Trans Neural Netw 17(5): 1301–1315. 17001989 10.1109/TNN.2006.875996

[62] Luo FQ, Wu JS, Yan K (2010) A Novel Nonlinear Combination Model Based on Support Vector Machine for Stock Market Prediction. Proceedings of the 8^th^ World Congress on Intelligent Control and Automation, Jinan, pp 5048–5053.

[63] Saigal S, Meheotra D (2012) Performance comparison of time series data using predictive data mining techniques. Advances in Information Mining 4(1): 57–66.

[64] Achelis S (2013) Technical Analysis from A to Z, 2nd Edition. McGraw-Hill.

[65] Plerou V, Gopikrishnan P, Rosenow B, Amaral LAN, Guhr T, et al (2002) A Random Matrix Theory Approach to Cross-Correlations in Financial Data, Phys. Rev. E 65 066126 10.1103/PhysRevE.65.066126 12188802

[66] Hong ZQ, Yang JY (1999) Optimal discriminant plane for a small number of samples and design method of classifier on the plane. Pattern Recognition 24(4): 317–324.

[67] Pinquier J, Karaman S, Letoupin L, Guyot P (2012) Strategies for multiple feature fusion with Hierarchical HMM: Application to activity recognition from wearable audiovisual sensors. 21st International Conference on Pattern Recognition (ICPR). Tsukuba, pp 3192–3195.

[68] Lendasse A, Lee J, Bodt ÉD, Wertz V, Verleysen M, et al.(2001) Dimension reduction of technical indicators for the prediction of financial time series - Application to the BEL20 Market Index. European Journal of Economic and Social Systems15N° 2 (2001) 31–48.

[69] Kwak N, Kim C, Kim H (2008) Dimensionality reduction based on ICA for regression problem. Neurocomputing 71(13–15): 2596–2603.

